# The yin-yang of immunity: Immune dysregulation in myelodysplastic syndrome with different risk stratification

**DOI:** 10.3389/fimmu.2022.994053

**Published:** 2022-09-23

**Authors:** Xiaohuan Peng, Xiaofeng Zhu, Tianning Di, Futian Tang, Xiaojia Guo, Yang Liu, Jun Bai, Yanhong Li, Lijuan Li, Liansheng Zhang

**Affiliations:** ^1^Department of Hematology, Lanzhou University Second Hospital, Lanzhou University, Lanzhou, China; ^2^Key Laboratory of the Hematology of Gansu Province, Lanzhou University Second Hospital, Lanzhou University, Lanzhou, China; ^3^Key Laboratory of the Digestive System Tumors of Gansu Province, Lanzhou University Second Hospital, Lanzhou University, Lanzhou, China

**Keywords:** myelodysplastic syndrome, immune dysregulation, Yin-Yang theory, different risk stratification, immunotherapy

## Abstract

Myelodysplastic syndrome (MDS) is a heterogeneous group of myeloid clonal diseases with diverse clinical courses, and immune dysregulation plays an important role in the pathogenesis of MDS. However, immune dysregulation is complex and heterogeneous in the development of MDS. Lower-risk MDS (LR-MDS) is mainly characterized by immune hyperfunction and increased apoptosis, and the immunosuppressive therapy shows a good response. Instead, higher-risk MDS (HR-MDS) is characterized by immune suppression and immune escape, and the immune activation therapy may improve the survival of HR-MDS. Furthermore, the immune dysregulation of some MDS changes dynamically which is characterized by the coexistence and mutual transformation of immune hyperfunction and immune suppression. Taken together, the authors think that the immune dysregulation in MDS with different risk stratification can be summarized by an advanced philosophical thought “Yin-Yang theory” in ancient China, meaning that the opposing forces may actually be interdependent and interconvertible. Clarifying the mechanism of immune dysregulation in MDS with different risk stratification can provide the new basis for diagnosis and clinical treatment. This review focuses on the manifestations and roles of immune dysregulation in the different risk MDS, and summarizes the latest progress of immunotherapy in MDS.

## 1 Introduction

“Yin-Yang theory” is an advanced philosophical thought in ancient China, and it is also the earliest naive materialism. Yin and Yang refer to two opposite aspects of interrelated things or phenomena in the natural world, and contain the concept of unity of opposites (1). In general, everything that is active, external, ascending, warm, bright and hyperactive belongs to Yang, while everything that is quiet, internal, descending, cold, dark and hypofunction belongs to Yin. The essence of the “Yin-Yang theory” includes four aspects: opposites between Yin and Yang, mutual rooting of Yin and Yang, waxing and waning of Yin and Yang, transformation between Yin and Yang (2). “Opposites between Yin and Yang” refers to the mutual restriction and struggle between Yin and Yang. “Mutual rooting of Yin and Yang” means that Yin depends on Yang and Yang depends on Yin. Neither side can exist independently of the other. This interdependent relationship is also known as “mutual root”. “Waxing and waning of Yin and Yang” means that Yin and Yang are not in a static state, but in a state of dynamic change. Waning of Yin will lead to waxing of Yang and vice versa. “Transformation between Yin and Yang” refers to either Yin or Yang may transform into its opposite side in given conditions. If we regard the waning and waxing relation between Yin and Yang is a process of quantitative change, then the inter-transformation between Yin and Yang is a qualitative change.

There seems to be some concepts in common between “Yin-Yang theory” and “ Immunology “. Specifically, the view of “Yin-Yang theory” is that the immune system is a unity of opposites between Yin and Yang, and it is necessary to keep relative balance between Yin (immune suppression) and Yang (immune hyperfunction), so as to fully play the role in the normal function (3). Immune hyperfunction of body such as the hypersensitive reaction of immunocompetent cells can be defined as the disease of “Yin waning and Yang waxing” in the traditional Chinese medicine, and its typical diseases are autoimmune disorders (AD) and various allergic reactions. On the contrary, immunodeficiency such as immune cell deficiency can be regarded the disease of “Yin waxing and Yang waning” in the traditional Chinese medicine, and its typical diseases are immunodeficiency diseases and tumors. The treatment of “adjusting Yin-Yang” in traditional Chinese medicine refers to that “reducing excess” is immunosuppressive therapy, and “supplementing insufficiency” is immunopotentiation therapy.

Myelodysplastic syndrome (MDS) is a heterogeneous group of myeloid clonal diseases originated from hematopoietic stem cells, with characteristics of dysplasia in the bone marrow (BM), ineffective hematopoiesis, refractory cytopenias and a high risk of transformation to acute myeloid leukemia(AML). The pathogenesis of MDS has heterogeneity and most patients have no clear etiological and causative factors. At present, the research on its pathogenesis involves multiple aspects including genetic abnormalities of endogenous hematopoietic stem/progenitor cells, epigenetic alterations, exogenous BM microenvironment changes and immune dysregulation. The dysregulation of immune and inflammatory signaling pathways in BM microenvironment plays an important role in the occurrence and development of MDS, which is also one of the research hotspots in recent years.

Revised international prognostic scoring system (IPSS-R) is one of the gold standard of risk stratification and prognostic assessment for MDS patients. According to IPSS-R, patients with lower-risk MDS (LR-MDS) are those with very low-risk, low-risk and some subsets of intermediate-risk (≤3.5 points), and patients with higher-risk MDS (HR-MDS) are those with some subsets of intermediate-risk (> 3.5 points), high-risk and very high-risk. With the deepening of the study on the immune pathogenesis of MDS, researchers found that the immune dysregulation in MDS with different risk stratification is different, and changes dynamically in the process of disease progression. The immune abnormalities of most LR-MDS such as the significant increase of cytotoxic T lymphocyte (CTL) and helper T cell 17 (Th17), and the significant decrease of regulatory T cells (Treg) suggest that the immune system is in an activated and pro-inflammatory state (Yang), resulting in an increase of apoptosis rate of hematopoietic stem cells (HSCs) (4). In addition, many patients with LR-MDS seem to benefit from immunosuppressive therapy. On the contrary, the immune system of most HR-MDS is in an inhibitory state (Yin), which makes a massive expansion of abnormal clone in BM microenvironment (5). Immune activation therapy including immune checkpoint inhibitors and tumor vaccines may prolong the survival for these HR-MDS patients. However, we should recognize that the immune dysregulation of some MDS (possibly mainly intermediate-risk patients) may be the coexistence of immune hyperfunction and immune suppression, which changes dynamically and transforms mutually in the process of development and treatment. Specifically, there may be immune suppression at a certain stage of LR-MDS so as to promote the development of LR-MDS to HR-MDS and even AML. There also may be immune activation during the process of development and treatment of HR-MDS, which makes HR-MDS good curative effect, even transforming to LR-MDS. In conclusion, the immune dysregulation of LR-MDS and HR-MDS can be summarized by an advanced philosophical thought “Yin-Yang theory” in ancient China. The balance of immune hyperfunction (Yang) and immune suppression (Yin) is constantly changing between LR-MDS and HR-MDS (3–5), and can transform to each other under certain conditions.

**Figure 1:**
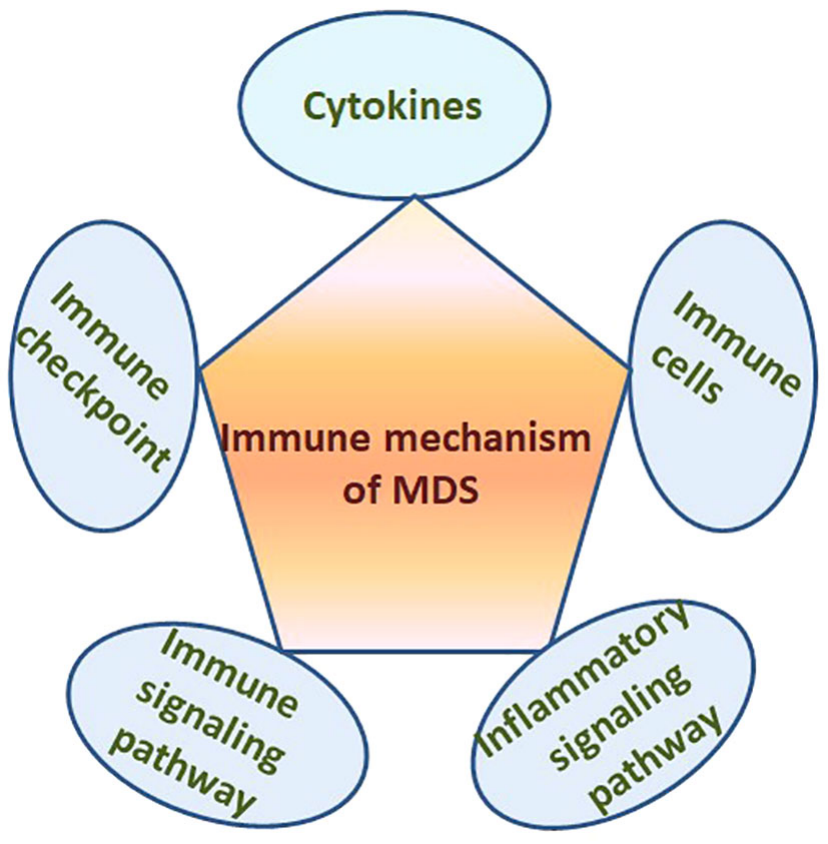
Many cytokines, immune cells, immune checkpoints, immune and inflammatory signaling pathways in the BM microenvironment participate in the pathogenesis of MDS.

It is well known that there is clear evidence of immune dysregulation in MDS patients. Many cytokines, almost all types of immune cells, immune checkpoints, immune and inflammatory signaling pathways participate in the pathogenesis of MDS (**Figure 1**). However, the manifestation and exact mechanism of immune dysregulation in MDS with different risk stratification are different and the immunotherapy plans should also be different. This review focuses on the different manifestations of immune dysregulation in MDS patients with different risk stratification, and summarizes the latest progress of relevant immunotherapy especially the emerging immunotherapy methods.

## 2 MDS and autoimmune disorders

Immune dysregulation and inflammatory reaction participate in the pathogenesis of MDS. Therefore, the relationship between AD which based on immune inflammatory response and MDS has also attracted the attention of the scholars. According to relevant researches, AD appears in around one third of MDS patients which was significantly higher than that of healthy people (6), while AD also increased the risk of MDS with an odds ratio (OR) from 1.5 to 3.5 (6, 7). The effect of AD on the clinical characteristics and prognosis to MDS patients is still controversial. In terms of clinical characteristics, the MDS patients with AD seems to be associated with female, lower hemoglobin levels and higher IPSS-R score (4, 7, 8) (**Table 1**). In terms of prognosis, most studies believe that AD has a positive or no effect on prognosis of MDS (**Table 1**) (8–14). Most AD associated with MDS can be efficiently managed with immune-therapeutic treatments. In conclusion, there is a clear relationship between AD and MDS, but its internal mechanism is not clear, and its potential prognostic impact is still controversial.

**Table 1:** The studies evaluated the frequency and characteristics of MDS patients with AD in the last 5 years.

Years Authors	Country	Ratio(n/N)	Main type of AD	Clinical features	Impact on survival	Reference
2021 Dongni Jiang et al	China	27.7%57/206	Vasculitis (19.3%, 11/57) Serum immune abnormality(17.5%, 10/57) RA (12.3%, 7/57)	Lower risk group;More MDS-MLD	Better PFSBetter OS	(9)
2021 Na Xiao et al	China	19.6%21/107	Vasculitis (23.8%,5/21) SLE (19.0%,4/21) RA (14.3%,3/21)	More MDS-MLDMore MDS-EB1	No difference	(10)
2019 Julie Seguier et al	France	11%88/801	Polyarthritis (27.2%,22/81) Immune cytopenias disorder(18.5%,15/81) Vasculitis (13.6%,11/81)	More MDS-MLDMore CMML-1	Better OS	(11)
2018 Montoro Jet al	Spain	48%68/142	Hypothyroidism (16.2%,11/68) RA (13.2%,9/68)Polymyalgia rheumatic (8.9%,6/68)	More FemaleLower hemoglobin value	Inferior OS	(12)
2016 Mekinian A et al	France	17.9%123/688	Vasculitis (32.0%,39/123) CTD (25%,31/123) Arthritis(23%,28/123)	More MDS-MLDMore MDS- EB1More CMML-1	No difference	(13)
2016 Komrokji RS et al	USA	27.8%391/1408	Hypothyroidism (44%,171/391) ITP (12%,46/391) RA (7%,28/391)	More FemaleLower RBC transfusion dependent	Better OSLess AML transformation	(8)
2016 Lee SJ et al	Korea	33.3%67/201	ND (35.8%,24/67) Behcet disease (14.9%,10/67) RA(13.4%,9/67)	More 5q- and +8	No difference	(14)

AD, autoimmune disease; AIM, autoimmune manifestation; MDS-MLD, Myelodysplastic syndrome-with multilineage dysplasia; PFS, free survival time; OS, overall survival; SLE, Systemic lupus erythematosus; RA, Rheumatoid arthritis; MDS-EB1, MDS with excess blasts 1; CTD, Connective tissue disease; ITP, Idiopathic thrombocytopenic purpura; RBC, Red blood cell; ND, Neutrophilic dermatosis.

Because the previous reviews had shown the relationship and mutual influence between AD and MDS, this paper will not review again in detail. This paper summarizes the relevant research results in recent 5 years in **Table 1** (8–14). At present, most studies believe that immune dysregulation is the common basis of the two diseases. Chronic immune stimulation may be the trigger factor for MDS, and some patients with MDS can get remission after immunosuppressive treatment, which provides evidence for this view.

## 3 Immune dysregulation of MDS

**Figure 2:**
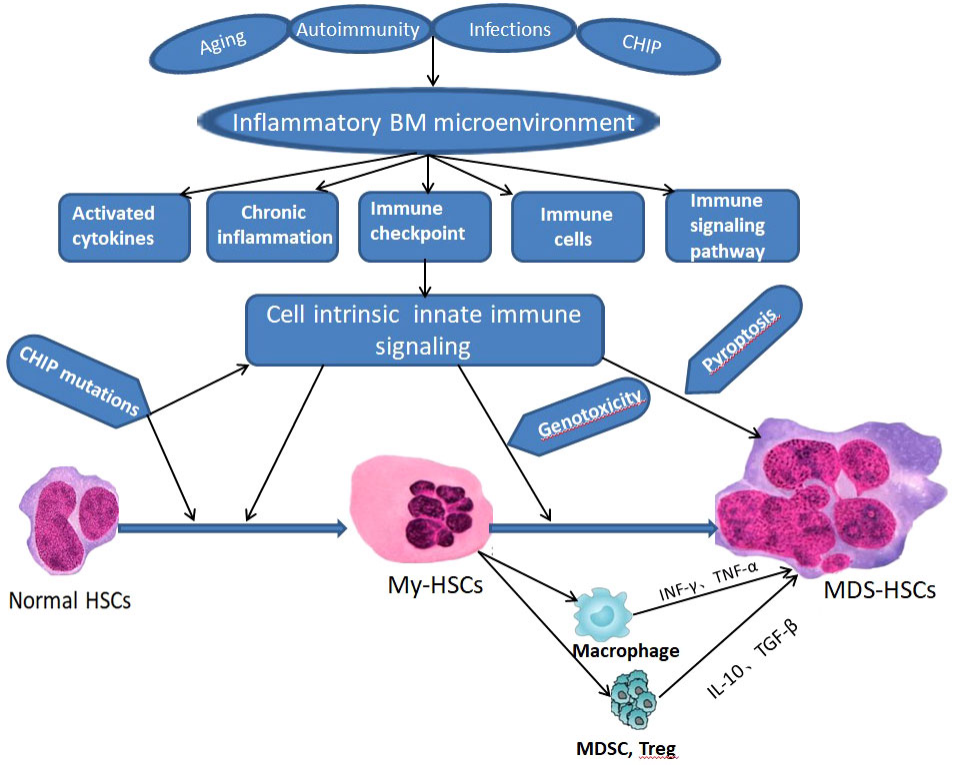
Schematic of innate immune signaling dysregulation in the pathogenesis of MDS. CHIP - clonal hematopoiesis of indeterminate potential, BM, bone marrow, HSCs - hematopoietic stem cells; My-HSCs, myeloid biased HSCs.

**Figure 3:**
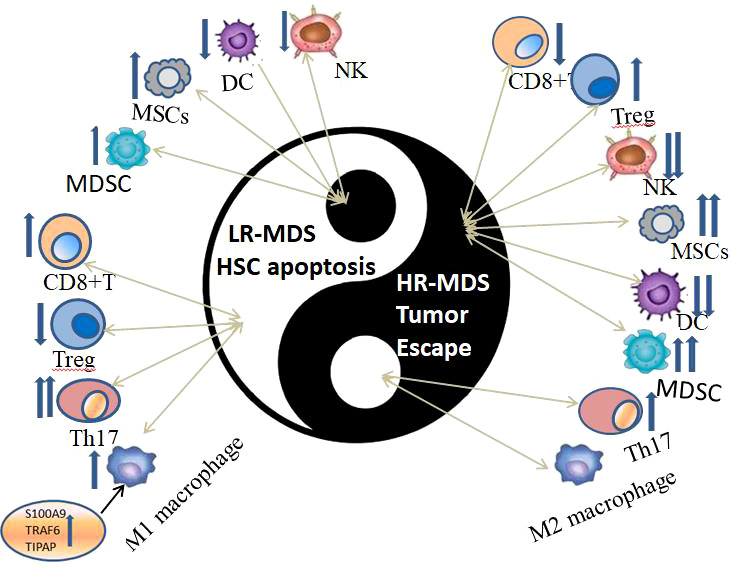
The immune dysregulation of immune cells in HR-MDS and LR-MDS can be summarized by an advanced philosophical thought “Yin-Yang theory” in ancient China, which are opposite, interrelated and can transform to each other under certain conditions.

Immune dysregulation in BM microenvironment plays an important role in the occurrence and development of MDS (**Figure 2**), which can be proved by the overexpression of TLR, CD14 and other immune related genes. However, the manifestations and intrinsic mechanisms of immune dysregulation in LR-MDS and HR-MDS are different. Cytokines, immune cells, immune checkpoints, immune and inflammatory signaling pathways play different roles in immune dysregulation of MDS with different risk stratification (**Figure 3**), which is itemized here below.

### 3.1 Cytokines

The abnormal expression of cytokines, chemokine and growth factors participate in the occurrence and development of MDS especially the abnormal secretion of cytokines. The levels of interferon-γ (IFN-γ), tumor necrosis factor-α (TNF-α), transforming growth factor-β (TGF-β), lnterleukin-6 (IL-6), IL-8, IL-32 and granulocyte macrophage colony stimulating factor (GM-CSF) generally increase in MDS patients, and their expression levels may be related to disease outcome (15, 16). Pardanani et al. found that the levels of 19 of the 30 plasma cytokines in MDS patients changed significantly, among which the increased levels of CXCchemokineligand-10(CXCL10), IL-7 and IL-6 seemed to be predictors of lower survival (16), while the increased levels of IL-4 and CCL3 were significantly correlated with higher remission rate (5). However, abnormal secretion of cytokine is different in MDS with different risk stratification.

In LR-MDS patients, the levels of pro-inflammatory cytokines such as TNF-α、IFN-γ、IL-8、IL-12 and IL-17 increase and induce the apoptosis of normal HSCs in BM (17, 18). In HR-MDS patients, myeloid-derived suppressor cells (MDSCs) from BM secrete a large number of immunosuppressive cytokines such as IL-10, IL-1β and TGF-β, causing tumor cells to escape from immune surveillance (19). IL-6 is an important regulatory factor of immune and inflammatory response, having different biological role and expression level in immune microenvironment in LR-MDS and HR-MDS. When being at low level, IL-6 mainly participates in clonal hematopoiesis of indeterminate potential (CHIP), hemocytopenia and BM hypoplastic of LR-MDS. When being at high level, IL-6 mainly participates in the tumor invasion, metastasis and recurrence of HR-MDS. So the overexpressed IL-6 is a predictor of lower survival and poor prognosis in patients with MDS (16, 20).

In addition, immunosuppressive cytokines (Yin) may also exist in immune microenvironment of some LR-MDS (Yang), and pro-inflammatory cytokines (Yang) may also be highly expressed in immune microenvironment of some HR-MDS (Yin). There may be a process of struggle between immunosuppressive cytokines and pro-inflammatory cytokines in these patients, which ultimately determines the development and outcome of the disease. Just like the “Yin-Yang theory”, two opposing immune status may also be interrelated and can transform to each other. For example, as a typical pro-apoptotic cytokine, TNF-α expression is generally increased in the peripheral blood of MDS patients and negatively correlated with IPSS-R prognostic score. So, TNF-α mainly participates in the occurrence and development of LR-MDS (18). It is worth noting that the level of TNF-α in some HR-MDS patient is also increased compared with healthy controls and may affect its disease development. The results confirms that immune activating molecules (Yang) have also increased in the immunosuppression microenvironment of HR-MDS (Yin), which is “Yin contains Yang” and “Yin generates Yang” in “Yin-Yang theory”. TGF-β is a pleiotropic cytokine, which is generally increased in the BM microenvironment of MDS and participates in the pathogenesis both of LR-MDS and HR-MDS. In LR-MDS, high level of TGF-β can directly enhance the p38-MAPK signaling pathway to promote the expression of pro-inflammatory genes of downstream and the differentiation of Th17 cells, increasing the expression of IL-17 and IFN-γ and finally inducing the occurrence and development of disease (21). In HR-MDS, high level of TGF-β from mesenchymal stem cells (MSCs) inhibits the normal function of B, T and NK cells and induces the proliferation of Treg cells, so as to promote the immunosuppressive microenvironment and development of disease (22). The influence of TGF-β to the immune status of MDS depends on the specific cell and microenvironment. It can not only promote apoptosis in LR-MDS (Yang), but also play an immunosuppressive role in HR-MDS (21, 22), and it also may mediate the mutual transformation of LR-MDS and HR-MDS under some conditions, reflecting the unity of opposites and mutual transformation of Yin and Yang. In addition, there are many dysregulated cytokines in the BM immune microenvironment of MDS, but the specific regulatory mechanism and impact on prognosis are still unclear, which needs further research.

### 3.2 Immune cells

#### 3.2.1 T lymphocyte subsets

T lymphocytes are the most important immune cells, and take part in both the cell-mediated immunity and humoral immunity at the same time. T lymphocytopenia is very common in MDS patients, but its manifestations are different in different risk stratification. CD8+T lymphocytes which are also called CTL are the effector T cell of anti-tumor immunity because they can directly kill tumor cells. CD8+T lymphocytes have been shown to activate and proliferate, inhibit malignant and normal HSCs hematopoiesis, and induce intramedullary apoptosis in LR-MDS patients (23). However, CD8+T lymphocytes show a significant decrease and induce the overexpression of programmed cell death protein 1/programmed cell death ligand 1 (PD-1/PD-L1) in the tumor microenvironment of HR-MDS patients, thereby enhancing the ability of tumor cells to escape host immunosurveillance (24). Treg cells are key regulators of immune system with strong immunosuppressive function and important for immune tolerance. Treg cells in BM and peripheral blood of LR-MDS patients are significantly decreased, and can be used as a prognostic factor to predict the degree of anemia, the rate of AML transformation and overall survival(OS) (25). On the contrary, the number and activity of Treg cells in HR-MDS are increased, which promotes MDS to escape immunosurveillance and transform to AML (26).

Th17 is originated from pluripotent CD4+T cells and mainly secretes pro-inflammatory cytokine IL-17. The levels of Th17 and IL-17 in BM and peripheral blood in LR-MDS patients are significantly higher than those in HR-MDS and healthy control group, which stimulate a variety of cytokines to produce inflammatory reaction, and finally lead to increased apoptosis of BM cells and ineffective hematopoiesis (17, 27). Previous study in our research group had got similar conclusions and proved that cyclosporine A, an immunosuppressive agent, can inhibit the function of Th17 cells to improve the morbid hematopoiesis of LR-MDS, further confirming that Th17 taken part in the immune hyperfunction of LR-MDS (28). Interestingly, although the levels of Th17 and IL-17 are significantly lower in HR-MDS than those in LR-MDS, they are higher than those in healthy control group (27). It suggests that there are also high expression of pro-inflammatory cell and cytokine (Yang) in the immunosuppressive microenvironment of HR-MDS (Yin), reflecting the concept of “Yin contains Yang” and “Yin generates Yang”.

Th22 cells are a newly discovered subset of helper T cells, which mainly secrete IL-22 and TNF-α. Based on the present studies, Th22 cells may have a dual immunomodulatory activity of pro-inflammatory and immunosuppression. For example, Shao et al. found that Th22 cells in peripheral blood of MDS patients increased significantly, and were significantly higher of HR-MDS than that of LR-MDS, indicating that Th22 cells may be more involved in immune escape (Yin) of MDS (29). However, another study found that number and effectors of Th22 cells in LR-MDS patients were higher than those in HR-MDS, suggesting that Th22 tends to pro-inflammatory characteristics (Yang) (30). Current studies have found that Th22 cells in MDS immune microenvironment may have both pro-inflammatory (Yang) and immunosuppressive (Yin) functions, may change dynamically, and even transform to each other under some conditions. Of course, it needs further research.

#### 3.2.2 Natural killer cells

NK cells are the first line of defense against antitumor immunity with direct killing effect and take part in both innate immunity system and adaptive immunity system. NK cells in peripheral blood and BM of MDS patients decrease significantly and show negative correlation with IPSS-R score (31, 32). In HR-MDS, the number and function of NK cells such as cracking tumor cells, secreting cytokines and proliferation *in vitro* are significantly damaged, consequently inhibiting the normal anti-tumor immune response and promoting disease progression (31). In LR-MDS, the number of NK cells is also decreased but higher than HR-MDS, and the existing NK cells seem to have increased activity and cytotoxicity to CHIP of MDS, thereby inhibiting disease progression (32). In addition, it observed that the number of NK cells in the BM immune microenvironment of LR-MDS patients is reduced, but the existing NK cells have stronger immune function. From the perspective of “Yin-Yang theory”, that is “Yang contains Yin” and “Yin contains Yang”.

Activating killer immunoglobulin-like receptors (aKIRs) are the most important molecules in regulating NK cell activation and function. In recent years, it has been observed that the number and haplotype of aKIR gene have changed in MDS patients, which affects its immune monitoring and prognosis. In 2015, Daher et al. first reported that the number of aKIR gene was associated with the risk stratification of MDS, and found that the number of it in HR-MDS patients was significantly lower than that in LR-MDS patients, but both of them were lower than that in healthy volunteers (33). Stringaris et al. found that the overexpressing aKIR haplotype A was significant correlation with the higher risk of AML transformation in MDS patients by further study, and may be an independent predictor of clinical outcome in MDS patients (34).

#### 3.2.3 Dendritic cell

DC is a important immunomodulatory factor, and the role of it in MDS has not yet been fully elucidated up to now. Current studies have shown that the number and the ability to activate T cells of mature and immature DC in MDS patients are significantly reduced, especially in HR- MDS (35), but the concrete effects of DC on the immune response for MDS patients with different risk stratification are different. The high level of pro-inflammatory factors such as IFN-γ and TNF-α in the BM immune microenvironment of LR-MDS (Yang) promote the maturation of reduced DC (Yin), and then fully mature DC which pulsed with antigens can induce the specific T cells to kill clonal MDS cells (Yang), resulting in the increased apoptosis of precursor cell (36). DC dysregulation in LR-MDS also reflects the view of “Yin-Yang theory”, that is “Yang contains Yin”, “Yin contains Yang”, “Yang generates Yin” and “Yin generates Yang”. In HR-MDS, the abnormality of DC is mainly characterized by the reduction of the number especially plasma like dendritic cells (pDCs), and the ability of DC to activate T cells is also significantly weakened (35, 37). In addition, whether HR-MDS or LR-MDS, the DC has obvious problem of differentiation and maturation. For example, the expression of some surface antigens such as CD54, CD80 and CD86 are reduced, and the ability to stimulate T cells and antigen presentation in mixed lymphocyte reaction is significantly reduced (38). In conclusion, all of the above studies illustrate the viewpoint of DC cell inefficiency in LR-MDS patients especially HR-MDS.

#### 3.2.4 Mesenchymal stem cells

MSCs are the key component of BM microenvironment in MDS patients and play an important role in maintaining the immune stability by down-regulating the intensity of immune response, regulating natural immunity and adaptive immunity. However, there is significant difference in the density and immune regulatory function of MSCs between LR-MDS and HR-MDS patients. The density of MSCs in HR-MDS patients is significantly higher than that in LR-MDS patients and has independent prognostic significance which is associated with lower OS and higher AML transformation rate (39, 40). MSCs of HR-MDS patients have immunosuppressive properties which are characterized by high level of TGF-β expression and the significantly enhanced ability to induce Treg and inhibit the proliferation and activation of T cells (5). There are great differences in the ability of MSCs to induce Treg between HR-MDS and LR-MDS. Compared with LR-MDS MSCs, HR-MDS MSCs can induce more Treg (40). It also reflects that immunosuppression from MSCs is more obvious in HR-MDS, although they also mildly displays immunosuppression (Yin) in the pro-inflammatory immune microenvironment of LR-MDS (Yang). In addition, the effect of MSCs on DC will also change dynamically with the disease state of MDS. The ability of MSCs to inhibit DC differentiation and maturation in HR-MDS is significantly better than LR-MDS MSCs (41). On the contrary, the ability of MSCs to inhibit DC differentiation and maturation is weak in BM immune microenvironment of LR-MDS (Yang), but there is still mild inhibition (Yin). Finally, it leads to the over activation of DCs with the strongest antigen-presenting function in LR-MDS which induce the excessive proliferation and activation of T cells in BM and then release a lot of pro-inflammatory molecules (Yang) to induce massive apoptosis of normal HSCs (40–42). In LR-MDS, the effect of MSCs on DC reflects that the “Yang contains Yin” and “Yin generates Yang” in “Yin-Yang theory”. In addition, some MSCs of HR-MDS (Yin) can promote the pro-inflammatory cytokines such as TNF-α and IFN-γ secretion (Yang) which induce the increase of PD-L1/2 synthesis and secretion, and finally inhibit the activation and proliferation of CD4+T cells and promote the apoptosis of T cells (Yin) (43). It is the “Yin contains Yang” and “Yang generates Yin” in “Yin-Yang theory”. In conclusion, all of these results suggest that MSCs have different immune regulation in different risk stratification MDS, which may be very important for understanding the pathogenesis of MDS and developing new immunotherapies.

#### 3.2.5 Myeloid-derived suppressor cells

MDSCs are special immune cells which are recently found and have inhibitory effects on the body’s immunity. MDSCs take part in the occurrence and development of MDS, but have different immunomodulatory effects in different risk. The number of MDSCs in LR-MDS patients is significantly lower than that in HR-MDS patients, which may be related to the immunosuppressive BM microenvironment of HR-MDS (44). In clinical practice, it is found that some LR-MDS (false Yang) will transform to HR-MDS (Yin), and the number of MDSCs will gradually increase in this process which may gradually induce the immune microenvironment of LR-MDS from activated state to inhibited state, that is “Yang generates Yin” and “Yin-Yang transformation” (45). Under the interaction of S100A9 and CD33, the BM microenvironment of HR-MDS drives the significant expansion of MDSCs and induces the immunosuppressive cytokines such as IL-10 and TGF-β overexpression. They inhibit the proliferation and function of T cells and NK cells, thus directly inhibiting normal hematopoiesis (46). MDSCs in HR-MDS can express CD155 to connect T cell immune receptor with immune checkpoint molecule T cell immunoglobulin and ITIM domain (TIGIT) and transmit inhibitory signal to NK cells, further aggravating the immunosuppressive microenvironment (46). In addition, high levels of monocytic MDSCs (M-MDSCs) in HR-MDS showed higher levels of intracellular IL-10, TGF-β and CXCR4 (45). In conclusion, MDSCs play an important role in the imbalance of immune monitoring of MDS and may be an important potential therapeutic target.

#### 3.2.6 Other immune cells

In addition to the above immune cells, other innate immune cells in MDS patients also have abnormal regulation, such as macrophages, monocytes, neutrophils and so on. In MDS, the number of macrophages is decreased and phagocytosis of macrophages is impaired. The study found that percentage of macrophages in BM of HR-MDS is significantly lower than that of LR-MDS (47). Macrophages in LR-MDS BM microenvironment are mainly the M1 type and secrete a variety of pro-apoptotic cytokines including TNF-α, inducing BM cell apoptosis. In addition, macrophages which highly express S100A8s can hinder the normal differentiation of erythrocytes in LR-MDS microenvironment (48). In HR-MDS, M2 macrophages are dominant in quantity and function, suggesting that high level of M2 macrophages may be an early warning index for the poor prognosis of MDS (49). Monocytes in MDS have a unique phenotype and can reduce the production of matrix metalloproteinase (MMP).which is an important secretion product and can inhibit the supporting role of BM microenvironment for HSCs (50). In addition, high level of MMP significantly inhibited erythrocyte proliferation which finally caused hemocytopenia, so monocytes mainly participate in the pathogenesis of LR-MDS (51). MDS derived tumor-associated neutrophils are the product of abnormal hematopoiesis and have functional defects which may eventually lead to high mortality of infection patients in HR-MDS (52).

### 3.3 Immune checkpoint

Immune checkpoints have become a research hotspot in recent years because of its unique immunosuppressive role in tumor-specific immunity, and had achieved a series of results. MDS cells have been proved to have ability to utilize the immunosuppressive effect of immune checkpoints to promote their survival and proliferation. Cytotoxic T lymphocyte-associated antigen-4 (CTLA-4) and PD-1/PD-L1 have been the most extensively studied immune checkpoints (53). In addition, many new immune checkpoint molecules including T cell immunoglobulin mucin-3 (Tim-3), lymphocyte activation gene-3 (LAG-3), CD47and TIGIT had also been successively proved to participate in the occurrence and development of MDS (54, 55).

As early as 2014, the University of Texas MD cancer center in the United States found that the expression of PD-1, PD-L1 and PD-L2 in CD34+ cells of MDS patients was significantly increased, which was related to the risk stratification of disease and the drug resistance mechanism of hypomethylating agent (HMA) (24). *In vitro* and animal experiments further showed that the BM microenvironment of MDS can induce the overexpression of immune checkpoint molecules such as PD-1/PD-L1 by activating MDSCs and specific cytokines (53, 56). The serum concentration of CTLA-4 in MDS patients increased too, and the HR-MDS group was significantly higher than LR-MDS. In addition, the overexpressed CTLA-4 in MDS is associated with high mortality (57). However, it can also be explained that CTLA-4, an important immunosuppressive molecule, can be used by tumor cells to induce immunosuppressive state and make tumor growth and development.

Tim-3 is a newly discovered immune regulatory molecule in recent years and combines with its ligand galectin 9 (Gal-9) to produce negative immune regulation, leading to Th1 cell apoptosis, IFN-γ release decreased and MDSCs proliferation. There are few studies on Tim-3 in MDS patients now, but the existing studies have confirmed that the expression of Tim-3 in BM of MDS patients is significantly higher than that of control group. The expression level of Tim-3 in low-risk group, medium-risk group and high-risk group increases successively, suggesting that Tim-3 may be a marker of malignant clones of MDS cells and participate in the malignant transformation of MDS (58, 59). In addition, the study found that the LAG3 expression on CD8+T and Treg cells in MDS patients was significantly higher than that in healthy controls. The overexpression of LAG3 may be the molecular basis for the low function of CD8+ effector T cells and the high function of Treg cells, so as to promote immune escape and eventually lead to disease progression (54). TIGIT is also a new immune checkpoint molecule, which is high expression on NK and T cells in MDS patients. TIGIT can directly inhibit the antitumor immune function mediated by NK and T cells and indirectly reduce the secretion of activated cytokines such as CD107a, IFN-γ and TNF-α to participate in disease progression and immune escape of MDS (55). As a new immune checkpoint molecule, CD47 on tumor cells is combined with signal-regulatory proteinα(SIRPα)to send “don’t eat me” signal to immune system, playing a key role in tumor cells recognition and immune escape, and gradually becoming an effective target of tumor immunotherapy. The studies have confirmed that MDS cells significantly overexpress CD47, which is associated with higher risk and poor OS (60). In conclusion, there is sufficient evidence to indicate that immune checkpoint molecules participate in the abnormal myeloid clonal response in MDS patients, which provides a new immunotherapy for HR-MDS. In addition, HMA treatment can significantly improve the expression of several immune checkpoint molecules on MDS cells such as PD-L1, Tim-3 and CD47, providing a theoretical basis for the mechanism of HMA resistance and the combination therapy with immune checkpoint molecular inhibitors (61).

Immune checkpoints are significantly overexpressed in HR-MDS patients, promoting the formation of immunosuppressive microenvironment (Yin), which is related to lower OS and higher AML transformation rate (57–60). Moreover, the studies had proved that immune checkpoint inhibitors can improve the prognosis of some HR-MDS patients and promote the recovery or enhancement the functions of immune active cells. From the perspective of “Yin-Yang theory”, it may be that HR-MDS immunosuppressive microenvironment (Yin) can also promote the production of immune active cells and molecules (Yang) and enhance the anti-tumor immune response under the certain conditions (such as immune activation treatment), that is “Yin generates Yang” and “Yin-Yang coexist”. In addition, the current study confirmed that the expression of immune checkpoint molecules such as PD-1/PD-L1 and CTLA-4 in some LR-MDS was also increased (24, 53). The results further suggest that immunosuppressive molecules are also expressed in the activated immune microenvironment of LR-MDS (Yang), there is “Yang contains Yin”. The expression of immune checkpoint molecules in some LR-MDS may be further increased under certain conditions such as the treatment of immunosuppressant, resulting in the gradual transformation of the activated immune microenvironment of LR-MDS to the inhibitory state (“Yin-Yang transformation”). Clinically, it is manifested as LR-MDS finally developed into HR-MDS, and immunosuppressant is ineffective in these LR-MDS patients.

### 3.4 Immune signaling pathway

Chronic innate immune and related inflammatory signaling pathways have been reported to play an important role in the pathogenesis of MDS for many years, but specific evidence has not been found until recently. Next, we will focus on the role of them in the occurrence and development of MDS in different risk stratification.

#### 3.4.1 Apoptosis signaling pathway

It is well known that abnormal apoptosis is an important factor in the pathogenesis of MDS. However, due to the heterogeneity of the disease, the different risk stratification MDS are affected by apoptosis differently, and LR-MDS is more closely related to apoptosis. Increased apoptosis was observed in LR-MDS, while apoptosis resistance was observed in HR-MDS. LR-MDS cells tend to pro-apoptotic phenotype, while HR-MDS cells changed to anti-apoptosis phenotype (62). As we all know that apoptosis is mediated by death receptor Fas and its specific ligand (Fas-L). In LR-MDS patients, TNF-α, Fas-L, TNF-Related apoptosis-inducing ligand receptor 1 (TRAIL-R1) and other pro-apoptotic cytokines are up-regulated, which promote the apoptosis of MDS clonal cells (63). The malignant clones with dysplasia in HR-MDS patients may produce resistance to pro-apoptotic effect of TNF-α, resulting in the increase of abnormal MDS clonal cells. In addition, CD34+ cells in HR-MDS show higher expression of anti-apoptotic gene Bcl-2 and lower apoptotic cell related antibody (Apo2.7), which explains why BM cells in HR-MDS are more resistant to apoptosis than those in LR-MDS.

Increased apoptosis is a unique characteristic of LR-MDS. Recent studies have found that in addition to the differential expression of apoptotic genes, a special inflammatory cell death process called pyroptosis may also contribute to apoptosis in LR-MDS (64). Reactive oxygen species produced by S100A9 and tumor necrosis factor receptor-associated factor 6 (TRAF6) can activate NLRP3 inflammasome, which eventually leads to the formation of pyroptosis in MDS patients and promotes hematopoietic failure of MDS (56). Inhibiting pyroptosis such as neutralizing S100A9, inhibiting NLRP3 and eliminating Caspase-1 have been shown to improve hematopoietic failure in MDS, providing new therapeutic prospects in MDS (65).

#### 3.4.2 Toll-like receptor signaling

Toll-like receptor (TLR) gene encodes key promoters of innate immune signal and plays a core role in innate immune response. The study found that more than 50% of MDS patients had overexpression of TLR signaling pathway and downstream effector molecules, including TLR-1, TLR-2, TLR-4, TLR-6, TLR-7, TLR-9 and its downstream effector molecules such as MyD88 or IRAK1 and IRAK4 kinases (66, 67). The enhanced TLR signaling is particularly significant in LR-MDS and leads to increased apoptosis and ineffective hematopoietic of the disease (66). In 2013, the University of Texas MD Anderson Cancer Center detected the mRNA expression of eight TLRs (TLR1-4 and TLR6-9) in HSCs of MDS patients and found that the TLR of LR-MDS was significantly higher than that of HR-MDS, especially TLR2 and TLR4 which were associated with increased apoptosis and better OS rate (67). Except for TLR, the mRNA expression of MyD88, a downstream molecule of TLR signaling pathway, is also increased, especially in LR-MDS, and blocking MyD88 can lead to increased erythroid colony formation (68). In addition, the levels of TLR4 ligands S100A8 and S100A9 in BM and peripheral blood of MDS patients especially LR-MDS are also increased. As an endogenous damage related mode molecule (DAMP), S100A8 and S100A9 can enhance the production of inflammatory components and pro-inflammatory cytokines by binding with TLR4, so as promoting the ineffective hematopoiesis of LR-MDS (69). In conclusion, the above studies show that TLR signal enhancement is a significant feature of MDS, especially LR-MDS. On the contrary, because the immune microenvironment of HR-MDS is inhibitory (Yin), so although the activated TLR signal (Yang) also plays a certain role in the pathogenesis of HR-MDS, it may not be able to resist the whole immunosuppressive microenvironment.

## 4 Immunotherapy for LR-MDS

Tumor immunotherapy is known as the most promising ways to cure cancer. Immune dysregulation plays an important role in the pathogenesis of MDS, so immunotherapy should also be one of the most promising treatments for MDS patients, and has achieved good clinical efficacy.

**Figure 4:**
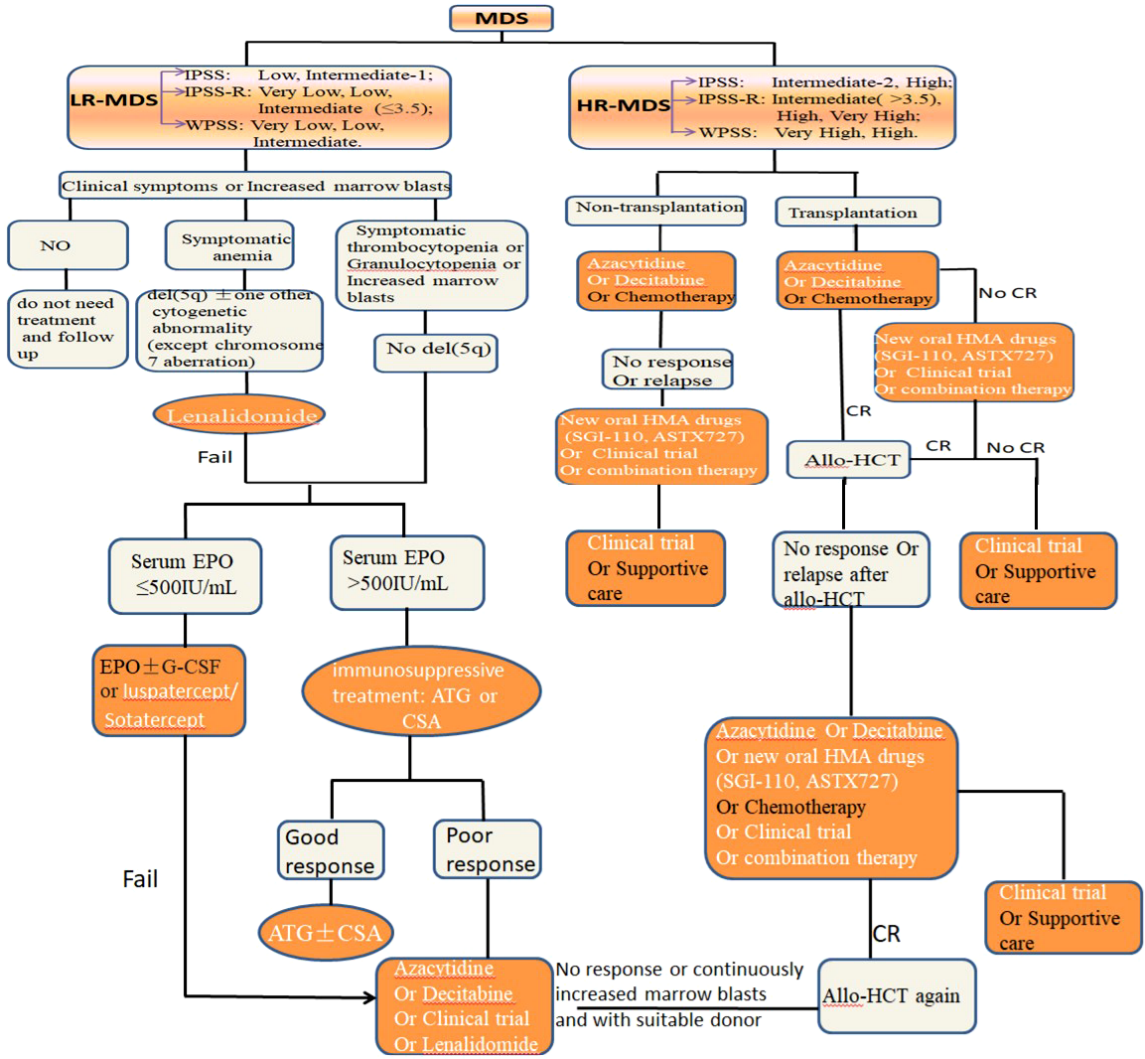
The standard treatment approach of MDS with different risk stratification, and immunotherapy is an important part (orange and white).

The “Yin-Yang of immunity in LR-MDS” shows us that immune system of most LR-MDS is in an activated and pro-inflammatory state, which leads to the increase of apoptosis. We can regard these LR-MDS as a disease of “Yang excess”. Therefore, immunosuppressant and immunomodulatory treatment will be a reasonable treatment for the disease. From the perspective of “Yin-Yang theory” of traditional Chinese medicine, it is described as “damaging its excess Yang” and “enriching Yin and suppressing Yang”. But some LR-MDS may be the coexistence of immune hyperfunction and immune suppression, so immunosuppressant treatment may accelerate the progression of the disease. So we should closely monitor the changes of immune indicators for these patients and provide precise immunotherapy. Next, we will review current application and prospect of immunotherapy in LR-MDS (**Figure 4**).

### 4.1 Immunosuppressant

The immune system of LR-MDS patients is in an activated and pro-inflammatory state, so immunosuppressant can reverse these immune responses to achieve the treatment effect. In clinical practice, immunosuppressants have been successfully used in LR-MDS patients for many years, and have achieved good clinical efficacy and safety (70, 71). A recent study found that the total effective rate of immunosuppressants in LR-MDS patients was 42.5%, of which the complete remission rate (CRR) was 12.5% and red blood cell transfusion independence rate was 33.4% (72). It was confirmed that immunosuppressants can successfully reduce the transfusion burden and related complications in LR-MDS patients. Antithymocyte globulin (ATG) and cyclosporin A (CSA) are the most commonly used immunosuppressants in the treatment of LR-MDS patients. ATG can reduce adaptive immunity to promote the recovery of hematopoietic function by consuming T cells and up-regulating Treg (70). CSA plays a role by inhibiting the expansion of cytotoxic T lymphocyte and inhibiting apoptosis related cytokines (71). When the ATG and CSA are combined, a long-term efficacy can be obtained (73). In addition, the use of immunosuppressants as the first-line treatment showed a better response rate than that as the third-line treatment (7). Immunosuppressants had no significant response in HR-MDS patients.

### 4.2 Monoclonal antibody

Some monoclonal antibodies have been proved to have therapeutic effect in LR-MDS because of their strong immunosuppressive effect, and Alemtuzumab is one of them. Alemtuzumab is a monoclonal antibody against CD52, which is mainly located on the surface of mature lymphocytes and weakens adaptive immunity by depleting lymphocytes. Therefore, Alemtuzumab can be used as an attractive alternative therapy for immunosuppressants. The latest study found that using Alemtuzumab to treat LR-MDS can achieve 68% hematological improvement or complete remission (CR), with a median remission period of 30 months (74).

Increased apoptosis is one of the characteristics of LR-MDS, so the treatment targeting for pro-apoptotic cytokines may be beneficial. TNF-α is an important pro-apoptotic cytokine in LR-MDS, so the monoclonal antibodies targeting for TNF-α such as Infliximab and Etanercept may improve prognosis in theory. However, the results of clinical trials showed that Infliximab alone had low activity and poor treatment response in LR-MDS (75). Another phase II clinical trial showed that the total effective rate of Etanercept combined with ATG in the treatment of LR-MDS was 56% (76). Anyways, the application of TNF-α inhibitors in LR-MDS patients has not achieved ideal results. But the TNF-α inhibitors have certain beneficial effects on BM inflammatory indexes, and the combination with other specific therapies may bring good news for LR-MDS patients.

TGF-β has been proved that it can participate in the occurrence of LR-MDS by promoting expression of pro-inflammatory genes, so inhibiting TGF-β is regarded as one of the potential treatments. Sotatercept (ACE-011) is a TGF-β ligand trap and has produced 49% of the total effective rate in a phase II clinical trial and can effectively improve anemia symptoms (77). Luspatecept (ACE-536) is new TGF-β Inhibitors. In a phase I/II clinical trial, 63% of LR-MDS patients has good therapeutic response and tolerability to ACE-536. Food and drug administration (FDA) has approved it to treat adult LR-MDS patients (78). Using TGF-β inhibitors may be beneficial for these patients who have few available treatments.

### 4.3 Novel immune pathway inhibitors

As mentioned earlier, TLR signaling pathway plays an important role in LR-MDS and leads to ineffective hematopoietic of the disease. Therefore, the use of TLR signaling pathway inhibitory drugs can improve the prognosis of LR-MDS in theory. At present, many related drugs have being undergone preclinical trials. For example, the TLR2 inhibitor OPN-305 has passed the phase I trial of healthy subjects and is currently being tested in the phase I/II trial of MDS patients (NCT02363491) (79).

### 4.4 Immunomodulatory drugs

IMIDs represented by “Lenalidomide and Thalidomide” have been proved to be beneficial and safe in patients with low or medium risk, single 5q deletion (del (5q)) and transfusion dependent MDS (80). Thalidomide mainly stimulates T cells, monocytes and inhibits the pro-inflammatory cytokine expression such as TNF-α, IL-12, IL-1 and IL-6 to play an immunomodulatory role. Thalidomide can improve erythropoiesis and prolong the time of non-transfusion-dependence, but may have obvious neurotoxicity and other toxic and side effects for some LR-MDS patients (81). Therefore, Lenalidomide which was less toxic but more effect was synthesized. Lenalidomide not only has many similar immunomodulatory effects to Thalidomide, but also can induce ubiquitination of specific substrates, degrade casein kinase 1 alpha 1, selectively inhibit the del(5q) MDS cells and reverse the abnormality of karyotype (82). As early as 2006, Lenalidomide has been approved by FDA to treat anemia of low-risk or medium-risk MDS patients with transfusion-dependent and del(5q), whether with or without additional cytogenetic abnormalities.

Lenalidomide is effective to treat del (5q) MDS patients, but more and more evidence supports its sensitivity of Lenalidomide to non del (5q) LR-MDS. National Comprehensive Cancer Network (NCCN) has recognized that Lenalidomide has a certain clinical efficacy in patients with non del (5q) MDS after failure of erythropoiesis stimulating agent (ESA) treatment, with a response rate of 43%, and more than a quarter of patients have achieved blood transfusion independence (83). In addition, the latest research indicates that when Lenalidomide is used before Azacytidine, a higher rate of hematological improvement can be obtained for patients with non del (5q) MDS and fail ESA treatment (84).

### 4.5 Hypomethylating agent

As a specific DNA methyltransferase inhibitor, HMA can inhibit abnormal DNA methylation and have shown good curative effect for MDS patients in clinical trials and practical applications. However, in addition to the direct cytotoxicity and demethylation to cancer cells, HMA also has epigenetic regulation. HMA can promote the gene expression of anti-tumor immunity, enhance tumor immunogenicity, and stimulate a variety of immune cells including macrophages, NK cells and CD8+T cells to secrete cytokines to exert cytotoxic effects and promote tumor cells death (85). HMA can also induce autologous antitumor immune response by consuming MDSCs (86). Azacytidine can also inhibit Treg proliferation and produce a large amount of IL-17, thus playing an immunomodulatory role (87). All kinds of evidence show that HMA has the function of immune regulation by affecting epigenetic.

At present, HMA is a ideal treatment drugs for HR-MDS and AML, but based on its apparent immunomodulatory characteristics, it can also be a good choice for non del (5q) LR-MDS patients who do not respond to first-line and second-line treatment (88). In fact, HMA has been approved for the treatment of LR-MDS in the United States and Japan and got 60% hematological improvement. CC-486 is a new oral preparation of Azacytidine which produces an encouraging total effective rate of 38% in LR-MDS and has good tolerance and safety. The most common adverse events of CC-486 are neutropenia, anemia and gastrointestinal disorders (89). The authors think that LR-MDS patients who do not respond to first-line and second-line treatment may be “false LR-MDS”, which is very likely to transform into HR-MDS. Therefore, HMA is effective for these patients.

In conclusion, immunosuppressive therapy and immunomodulatory therapy have a good effect to some LR-MDS patients, which can improve its hematological symptoms and morbid hematopoiesis. It accords with the activated immune microenvironment of LR-MDS. However, we should also recognize that nearly half of LR-MDS patients have poor or even ineffective effects on various immunosuppressive therapy and immunomodulatory therapy (74), while HMA drugs have a certain efficacy for these patients (90, 91). From a clinical point of view, we highly suspect that these LR-MDS tend to be “false LR-MDS”, and their clinical characteristics and prognosis are more inclined to HR-MDS. It also suggests that there may also be immunosuppressive factors (Yin) in the activated BM immune microenvironment (Yang) of LR-MDS and immune activation therapy can also improve the prognosis of some LR-MDS patients, reflecting the “Yang contains Yin” and “Yang generates Yin” again.

## 5 Immunotherapy for HR-MDS

Different from the immune state of LR-MDS, the immune microenvironment of HR-MDS is in an inhibitory state, which makes CHIP dramatically expand and escape immune surveillance. Traditional Chinese medicine regards this kind of immune disease as “Yin excess and Yang deficiency disease”, so immune activation therapy will be a reasonable treatment for HR-MDS. From the perspective of “Yin-Yang theory”, it is “supplementing its deficiency” and “supporting Yang and suppressing Yin”. But, there are also a few HR-MDS who may be the coexistence of immune hyperfunction and immune suppression, so immunosuppressant treatment may produce unexpected therapeutic effects for these patients. Next, we will review the current application and prospect of immunotherapy in HR-MDS (**Figure 4**).

### 5.1 HMA

From the above, we can know that HMA has immunomodulatory effect and is the main drug of the first-line treatment for HR-MDS patients. The initial response of HMA in the treatment of HR-MDS is good, but 40% of patients will become resistant to Decitabine and Azacytidine. So, there is an urgent need to explore new HMA drugs to reduce resistance. Guadecitabine (SGI-110) is a new type of HMA. The phase II clinical trial of HR-MDS patients with Azacytidine resistance showed that objective response rate was 14.3%, and the survival time of responders was significantly improved (NCT02197676) (90). ASTX727 is also a new oral HMA, which was approved by FDA in 2020 for new and secondary MDS with specific FAB subtypes (RA, MDS-RARS, MDS-RAEB and CMML) and IPSS score (middle-1, middle-2 and high risk). The studies have confirmed that oral ASTX727 and intravenous infusion of Decitabine have similar area under the concentration-time curve, safety, clinical response and lower drug resistance (91).

The mechanism of HMA resistance in MDS patients has not been fully clarified, but the up-regulated expression of immune checkpoint molecules may play a certain role. While enhancing the anti-tumor immune response, HMA also up-regulates the expression of immune checkpoint molecules, inhibits and even depletes the tumor specific T cells, resulting in tumor immune escape (61). Therefore, it seems very interesting to evaluate the efficacy of HMA combined with immune checkpoint inhibitors in HR-MDS patients, as shown below.

### 5.2 Immune checkpoint inhibitor

As we all know, immune escape is an important feature of HR-MDS, and one of the main mechanisms is the up-regulation of immune checkpoint molecules in BM microenvironment. Therefore, immune checkpoint inhibitor is a reasonable treatment strategy for HR-MDS. CTLA-4 and PD-1 inhibitors have been shown to play a role in HR-MDS by blocking the inhibitory signals on T cells and stimulating antitumor immune response (92–97). In addition, some new immune checkpoint inhibitors such as Tim-3 and CD47 inhibitors are also actively being conducted in various clinical trials.

#### 5.2.1 PD-1/PD-L1 inhibitors

The expressions of PD-1, PD-L1 and PD-L2 were significantly increased in MDS patients, and were related to the risk stratification and the drug resistance mechanism of HMA (24). Therefore, PD-L1/PD-1 is an ideal therapeutic target for HR-MDS patients, and PD-L1/PD-1 inhibitors may be potential drugs for recurrent and refractory MDS. Pembrolizumab is a humanized anti-PD-1 monoclonal antibody (98). The phase 1b clinical trial show that the OS of Pembrolizumab in MDS patients with HMA refractory is 6.0 months, the 2-year total OS rate is 17% and has controllable safety and clinical activity (NCT01953692) (92). Single PD-1 inhibitors have certain efficacy in the treatment of HR-MDS patients failed by HMA, but the effect is limited. Therefore, many scholars put forward a new viewpoint of “PD-1 inhibitors combined with HMA”. The latest phase 2 clinical trial showed that the objective remission rate (ORR) of Azacytidine combined with Pembrolizumab in the treatment of newly diagnosed HR-MDS patients was 76%, the CR rate was 18%. The ORR rate of Azacytidine combined with Pembrolizumab to HR- MDS patients who failed in the treatment of HMA was 25%, and the CR rate was 5% (93). In addition, a phase 1 study found that nivolumab, a PD-1 inhibitor, had good curative effect for relapsed HR-MDS after allogeneic transplantation (99). PD-1 inhibitor combined with HMA has a certain effect in HR-MDS patients, which makes some researchers focus on “PD-L1 inhibitor combined with HMA”. In 2022, the latest clinical trial published on “Blood” found that the ORR of PD-L1 inhibitor Durmalumab combined with Azacytidine in the treatment of HR-MDS was 61.5%, the ORR of Azacytidine alone was 47. 6%, but Durmalumab combined with Azacytidine had more toxic than Azacytidine alone (NCT02775903) (94). Atezolizumab is a new type of PL-L1 inhibitor and Guadecitabine (SGI-110) is a new type of HMA. In the phase 2 clinical trial, the combination of them in the treatment of relapsed refractory HR-MDS can get 33% ORR and prolong the survival for some HR-MDS patients (NCT02197676) (90). In general, these clinical trials show that the PD-1/PD-L1 inhibitor may have better antitumor activity and safety in some patients. The curative effect is more obvious in primary HR-MDS patients, and it also has a certain curative effect in patients failed by HMA, which is worthy of further research. In addition, many clinical trials of PD-L1/PD-1 inhibitors in the treatment of HR-MDS are in progress (**Table 2**) (90, 93, 94, 99, 100), which bring hope to patients.

**Table 2:** Clinical trials of immune checkpoint inhibitor in MDS.

ImmuneCheckpoint	Drug	Phase	Status	YearReported	IPSS Risk Category	Outcomes	Conclusion	Clinical Trial Identifier
PD-1	Pembrolizumab	Ib	Completed	2016	Int**-**1 and Int**-**2 and High;HMA failure:28	ORR=14-25% ( 1PR)	Manageable safety profile and potential activity	NCT01953692
	Pembrolizumab+AZA	II	Recruiting	2019	Int-1 andInt**-**2 andHigh;HMA failue:20,MDSfrontline:10	HMA failue: ORR=30%,MDSfrontline: ORR = 70%	Relatively safe and well-tolerated,mayhave antitumor activity	NCT03094637
	Nivolumab	I/Ib	Active not recruiting	2020	High; post-HSCT relapse of MDS:7	ORR=43%	Moderate antitumor activity but severe GVHD and irAEs	NCT01822509
	Nivolumab+AZA	II	Completed	2018	Int-1 and Int-2 and High;MDS frontline:20	ORR=75% (CR/CRp=50%)	Manageable safety profile and potential activity	NCT02530463
PD-L1	Durvalumab+AZA	II	Completed	2022	High;42 MDS	ORR = 61. 9%	No significant difference in safety and efficacy	NCT02775903
	Atezolizumab+Guadecitabine	I/II	Active, not recruiting	2018	Int-1 and Int-2and High;R/R MDS:9	ORR=33%(HI=22%,CR=11%)	Had an acceptable toxicity profile	NCT02935361
CTLA-4	Ipilimumab	I/Ib	Completed	2018	Int-1 and Int-2and High;HMA failue:29	ORR=7%	Safe but had limited efficacy as a monotherapy	NCT01757639
	Ipilimumab	II	Completed	2018	Int-1 and Int-2 andHigh;HMA failue:20	ORR=35%	Had limited efficacy as a monotherapy	NCT02530463
	Ipilimumab+AZA	II	Completed	2018	Int-1 and Int-2 and High;MDS frontline:21	ORR=71% (CR/CRp=38%)	Manageable safety profile and potential activity	NCT02530463
CTLA-4+PD-1	Ipilimumab+ Nivolumab	II	Recruiting	2018	Int-1 and Int-2 and High;HMA failue:8	ORR = 29%	Clinical activity could be seen in R/R MDS	NCT02530463
	Ipilimumab+Nivolumab+AZA	II	On Hold	2018	Int-1 and Int-2 and High;MDS frontline:6	ORR=50%(3 CR)	Had a better efficacy in frontline MDS	NCT02530463
TIM-3	MBG453+DEC	I/Ib	Recruiting	2020	High;MDS frontline:19	ORR=58%	Hada better efficacy andmanageable safety profile	NCT03066648
	MBG453 + AZA	I/Ib	Recruiting	2020	High;MDS frontline:13	ORR=70%	Had better efficacy and manageable safety profile	NCT03066648
CD-47	Magrolimab	Ib	Recruiting	Not Reported	Int-1 and Int-2 andHigh;R/R MDS:4	Not Reported	Not Reported	NCT03248479
	Magrolimab+ AZA	Ib	Recruiting	2020	Int-1 and Int-2 andHigh;MDS frontline:39	ORR= 91%	Had better efficacy and manageable safety profile	NCT03248479
	TTI-621 (SIRPαFc)	I	Recruiting	Not Reported	Not Reported	Not Reported	Not Reported	NCT02663518

ORR, overall reponse rate; Int-1, Intermediate-1; Int-2, Intermediate-2; CR, complete response; PR, partial response; CRp, complete remission with incomplete platelet recovery; CR/CRp, complete remission or complete remission with incomplete platelet recovery; AZA, Azacitidine; DEC, Decitabine; irAEs, immune-related adverse events.

#### 5.2.2 CTLA-4 inhibitor

As mentioned earlier, MDS cells have been shown to overexpress CTLA-4 which is associated with poor prognosis. Therefore, inhibiting CTLA-4 is also one of the potential treatments for HR-MDS. Lpilimumab is a humanized monoclonal antibody against CTLA-4 (101). A phase 1b clinical trial showed that Ipilimumab alone to treat HR-MDS patients failed by HMA can get 21% clinical benefit rate (clinical benefit rate is defined as disease stable condition for more than 12 months) and increase the number of effector T cells (95). Subsequently, Garcia Manero et al. reported the results of phase II clinical trials of Ipilimumab to treat patients failed by HMA and newly treated HR-MDS. It was found that Ipilimumab alone for HR-MDS patients failed by HMA can get an ORR of 35% and the combination of Ipilimumab and Azacytidine for newly treated HR-MDS patients can get an ORR of 71% (96). Above clinical trials show that the efficacy of Ipilimumab alone is limited, and the combination of HMA and Ipilimumab is better, but it still needs further research.

#### 5.2.3 Anti-Tim-3 monoclonal antibody

Tim-3 is a newly discovered negative molecule of immune regulation in recent years. The study found that the ligand of Tim-3 is preferentially overexpressed on leukemia and MDS HSCs compared with normal HSCs (58). This discovery eventually led to the production of anti-TIM-3 monoclonal antibody, and Tim-3 has become a possible new therapy way for HR-MDS. MBG453 is a new anti-Tim-3 monoclonal antibody. The results of phase 1 clinical trial showed that MBG453 combination with Decitabine has achieved 50% CR and molecular CR (MCR) in HR-MDS patients (97). Another phase 1b clinical trial conducted by Brunner et al. has reached similar conclusions (102). In addition, the immune related adverse events of MBG453 in the above clinical trials were low, and only one patient had elevated liver enzymes (grade 3). More relevant clinical trials are also in progress, as shown in **Table 2**.

#### 5.2.4 Anti-CD47 monoclonal antibody

CD47 is significantly overexpressed in MDS patients and can combine with the receptor SIRP-α to prevent macrophages from phagocytizing MDS cells (103). Therefore, CD47 has also become a new target for HR-MDS, and anti-CD47 monoclonal antibodies such as Magrolimab, TTI-621 and CC-90002 were finally introduced into the treatment. Magrolimab, also known as Hu5F9-G4, is a humanized anti-CD47 monoclonal antibody. A phase 1b trial of Magrolimab combined with Azacytidine in the treatment of HR-MDS reported 54% CR with good tolerance and safety (104). TTI-621, also known as SIRPα-IgG1 FC, is a unique SIRPαFc decoy receptor, which can target CD47 and block its activity and has been shown to be effective against recurrent or refractory hematological malignancies (105). A clinical trial to evaluate the efficacy of TTI-621 in the treatment of HR-MDS has entered phase I (NCT02663518) (105). CC-90002 is another humanized anti-CD47 monoclonal antibody. The results of phase 1 clinical trial in relapsed or refractory HR-MDS showed that it had poor efficacy and serious treatment-related side effects, which eventually led to the cessation of relevant clinical trials (106).

TIGIT, LAG3 and other immunosuppressive factors are also highly expressed in HR-MDS patients and the potential immunotherapeutic targets of HR-MDS in theory. However, there is no clinical trial using relevant monoclonal antibody now. In conclusion, the current use of immune checkpoint inhibitor in the treatment of MDS is still in its early stage, and more clinical trials are still needed to evaluate the safety, efficacy, optimal timing and potential combination therapy methods (107).

### 5.3 Adoptive T-cell transfer therapy

Adoptive immunotherapy is an important part of tumor immunotherapy, and adoptive T-cell transfer therapy is one of the important components. The three adoptive T-cell transfer therapy represented by chimeric antigen receptor T (CAR-T) cell therapy, Tumor infiltrating lymphocytes (TIL) therapy and T cell receptor T cells (TCR-T) therapy provide a possibility of “theoretical curative” for hematological tumors. Current studies have confirmed that T-cell transfer therapy has a good effect for lymphoma, but there are few studies on myeloid malignancies.

CAR-T cell therapy is the most common and hot adoptive T-cell transfer therapy. The clinical trials have preliminarily explored its role in MDS and achieved promising results. Finding suitable target antigen is the most critical link in CAR-T cell therapy. Natural killer type 2 receptor (NKG2D) is a positive immunomodulatory protein on NK and CD8+T cells, and is one of the ideal target antigens for CAR-T cell therapy in the treatment of HR-MDS. The phase I clinical trial of NKG2D-CAR-T cell therapy for HR-MDS is in progress (NCT02203825) and preliminarily report transient hematological improvement *in vitro* treatment, but further research is still needed (108). In 2019, Steven et al. found that the cell surface antigen CD123 was overexpressed on MDS stem cells and was related to the MDS risk stratification. So they proposed that CD123 could be used as one of the targets of CAR-T cell therapy for HR-MDS patients. It was subsequently confirmed that CD123 CAR-T cells could root out CD123+MDS stem cells *in vitro* (109). In addition, early preclinical data showed that CD123 CAR-T cell therapy can eliminate abnormal clones of MDS in derived xenotransplantation model (110).

### 5.4 DC-targeted immunotherapy

The ability to stimulate T cells and antigen presentation in MDS patients is significantly reduced, which makes DC cells become the new target of immunotherapy. The anti-tumor immunotherapy targeting DC cells is DC vaccine. Monocyte-Derived DC (mo DC) and leukemia derived DC (DCleu) are the main DC vaccines used for AML and MDS so far. Studies have confirmed that the Mo DC vaccine loaded with leukemia associated antigen (LAA) can effectively promote the apoptosis of HR-MDS and AML cells, and may produce good results for these patients (111). Christian et al. found that Mo DC/DCleu vaccine can activate the innate and adaptive immune system especially leukemia specific T cells, and enhance the killing effect on leukemia cells (112). In conclusion, treatment based on DC/mo-DCC/DCleu may be a promising way for HR-MDS patients, but continuous research including animal and human trials must be carried out.

### 5.5 NK cell adoptive transfer therapy

With the success of adoptive cell immunotherapy in tumor immunotherapy, NK cells have attracted more and more attention in cell adoptive therapy because they do not need pre-sensitization and will not lead to graft versus host disease. Then NK cell adoptive transfer therapy appears. NK cell adoptive transfer therapy is a new research field, which has shown a certain effect in AML and HR-MDS. *In vitro* experimental studies found that NK cells whether from umbilical cord blood, paired donors or autologous collection can expand in the presence of K562 leukemia cell line and produce strong tumor cell killing effect (113). The *in vivo* experiments in 16 patients with recurrent refractory MDS or AML also found that NK cell adoptive transfer therapy had good efficacy and tolerance, which further proved that HR-MDS would respond to adoptive transfer therapy, and supported the NK cell infusion as a bridge treatment before HSCT in refractory MDS or AML (114). Although it is confirmed that targeting NK cells may be a potential and effective therapy for HR-MDS patients, there are few relevant studies on NK cell adoptive transfer therapy, and further research is still needed.

### 5.6 Vaccine treatment

Wilms’ tumor 1(WT1) is a tumor suppressor gene located on chromosome 11p13. The immunotherapy targeting WT1 has been proved to induce the immune system to produce memory T cells and effector T cells which has the functions of immune monitoring and immune killing. Studies have confirmed that WT1 is overexpressed in CD34+MDS/AML stem cells and is associated with a higher blast cell counts and a lower OS (115). Therefore, WT1 can be used as the first target antigen for vaccine treatment for HR- MDS. The phase I clinical trial conducted by Tawara et al. found that WT1-specific T-cell receptor gene-transduced lymphocytes had some safety and persistence in the treatment of AML and HR-MDS. All patients had clonal expansion of WT1-specific T-cell (mainly CD8+T cells) at different degrees, of which 5/8 patients continuously had WT1-specific T-cell (UMIN000011519) (116). WT4869 is a synthetic peptide vaccine. Suzuki et al. evaluated the safety and efficacy of WT4869 in 25 HR-MDA/AML patients. WT1-specific T-cell was observed in 11 patients, and the median OS reached to 55.71 weeks (CTI-101374) (117).

Another tumor vaccine target in HR-MDS is PR1 peptide, which is an HLA-A2-restricted peptide targeting myeloid tumor cells. It can be recognized by CTL, and then forms PR1 specific CTL (PR1-CTL) to mediate the specific lysis of AML and HR-MDS cells. Muzaffar et al. had studied the efficacy and tolerance of PR1 peptide vaccine in HR-MDS patients. The results showed that PR1 peptide vaccine can induce specific immunity reaction and related clinical reactions in MDS patients including molecular remission and no adverse autoimmunity symptoms, which finally lead to the increase of PR1-CTL in circulating (118). In addition, in order to evaluate the effect of the combined application of PR1 and WT1 peptide vaccine in HR-MDS, 8 patients were vaccinated with both PR1 and WT1 peptide vaccines at the same time. The results showed that the number of PR1 and WT1 specific T cells increased, and stable disease lasted for more than 2 years (NCT00499772) (119).

NY-ESO-1 is another antigen with high immunogenicity and expressed in a variety of tumors. It is also another candidate target antigen in MDS and AML vaccine trials. The latest study used HLA-unrestricted NY-ESO-1 vaccine combined with Decitabine to treat HR-MDS patients, and all patients showed NY-ESO-1 gene expression and induced NY-ESO-1 specific CD4+ and CD8+T cells (120). In conclusion, the current studies suggest that tumor vaccine has a certain efficacy in HR-MDS patients, but there are few relevant clinical studies. Future studies should further expand related clinical studies and explore the efficacy and safety of vaccines combined with HMA or immune checkpoint inhibitors in the treatment of HR-MDS, so as to induce deeper and more lasting clinical response.

In summary, the immunotherapy principle of HR-MDS is to actively find various targets to restore and enhance the number and function of anti-tumor immune cells and cytokines (Yang) in the BM immunosuppressive microenvironment (Yin). The current results of studies confirm that immune activation therapy does have a certain effect on some HR-MDS and can improve its prognosis. It is further confirmed that immunosuppressive microenvironment (Yin) of HR-MDS also contains the immunocompetent cells and can promote its proliferation (Yang) under certain conditions (such as immune activation therapy), that is the “Yin contains Yang” and “Yin generates Yang”.

## 6 Conclusions and perspectives

In the past decade, we have deeply recognized that (1): There is a close relationship between AD and MDS, and the immune dysregulation may be the common driving force for AD and MDS (2); Immune dysregulation are complex and heterogeneous in the occurrence and development of MDS. LR-MDS is immune hyperfunction and increased apoptosis, HR-MDS is immune suppression and immune escape, while some MDS change dynamically which is characterized by the coexistence and mutual transformation of immune hyperfunction and immune suppression (3); Immune dysregulation of MDS with different risk stratification can be summarized by an advanced philosophical thought “Yin-Yang theory” in ancient China, meaning that LR-MDS and HR-MDS are opposite to each other, have a balance of waning and waxing, depend on each other and may transform into its opposite side under given conditions; (4) Immunotherapy strategy has become one of the hotspots in the treatment of MDS in recent years, and have achieved good results in clinic. The present research difficulties and challenges are that (1) The internal mechanism and mutual influence of the close relationship between AD and MDS are not yet fully clear; (2) How to identify these MDS patients who may be transformed and the concrete time and condition of transformation in the process of clinical diagnosis and treatment is important; (3) Whether these MDS patients who may be transformed can receive immunotherapy and what kind of immunotherapy they should receive, how to monitor the relevant immune changes in the process of treatment, and how to adjust the immunotherapy plan in time; (4) In addition, immunotherapy can only be said to be a promising potential treatment at present because immune dysregulation of MDS have great heterogeneity, so how to explore more reasonable combination therapy to improve the clinical response rate and OS of MDS patients is very important.

Taken together, we think that a better understanding of the mechanisms and manifestations of immune dysregulation in MDS with different risk stratification can help us to provide a new breakthrough in the area of MDS immunotherapy, and more importantly that it will provide a scientific rationale for clinical trials. Future research should further explore the immune dysregulation status of MDS with different risk stratification, and actively explore the new combination therapy ways such as immunotherapy combined with HMAs, hoping to bring new hope to patients who fail the standard therapy of MDS.

## Author contributions

XP, LZ, and LL designed the paper and recommended a structure for the review. XP, LZ, LL, XZ and TD wrote the initial draft and prepared figures. FT, YLiu, XG, JB and YLi helped to revise the manuscript. All authors contributed to the article and approved the submitted version.

## Funding

This work has been supported by the Project entrusted by National Clinical Medical Research Center for Hematological Diseases (grant 2021WWA01), Innovation base and talent plan of Gansu Province (grant 21JR7RA435) and Cuiying Scientific and Technological Innovation Program of Lanzhou University Second Hospital (grant CY2019-MS14).

## Conflict of interest

The authors declare that the research was conducted in the absence of any commercial or financial relationships that could be construed as a potential conflict of interest.

## Publisher’s note

All claims expressed in this article are solely those of the authors and do not necessarily represent those of their affiliated organizations, or those of the publisher, the editors and the reviewers. Any product that may be evaluated in this article, or claim that may be made by its manufacturer, is not guaranteed or endorsed by the publisher.
